# Sodium fluxes and silicon at the root plasma membrane: a paradigm shift?

**DOI:** 10.1093/jxb/ery042

**Published:** 2018-03-24

**Authors:** Guillermo E Santa-María, Francisco Rubio

**Affiliations:** 1 Instituto Tecnológico Chascomús (INTECH), Consejo Nacional de Investigaciones Científicas y Técnicas, Universidad Nacional de San Martín (CONICET-UNSAM), Avenida Intendente Marino, Chascomús, Buenos Aires, Argentina; 2 Centro de Edafología y Biología Aplicada del Segura (CEBAS), Consejo Superior de Investigaciones Científicas (CSIC), Campus Universitario de Espinardo, Espinardo, Murcia, España

**Keywords:** Apoplast, bypass, cycling, efflux, influx, silicon, sodium, rice (*Oryza sativa*)

## Abstract

This article comments on:

**Flam-Shepherd R, Huynh WQ, Coskun D, Hamam AM, Britto DT, Kronzucker HJ.** 2018. Membrane fluxes, bypass flows, and sodium stress in rice: the influence of silicon. Journal of Experimental Botany **69,** 1679–1692.

**Sodium fluxes in the root are critically important in salt tolerance, but new research using rice and including silicon suggests we need to revise our current understanding.** Flam-Shepherd *et al.* (2018) **provide evidence supporting the hypothesis that the extent of Na influx and cycling at the plasma membrane is far lower than previously assumed, along with significant and potentially masking apoplastic Na cycling. Silicon exerts an important effect on Na concentration in the shoot, but it appears that this is not the case in the roots. It is a paradigm shift and poses many new questions for the field.**

Modern agriculture needs to increase production to feed an expanding world population, expected to be almost 10 billion by 2050. However, success is threatened by salinity, an abiotic stress frequently produced by high external sodium and chloride concentrations in the soil. This stress reduces crop yield and quality and affects more than 20% of irrigated land, a figure likely to be exacerbated by global climate change (Munns and Tester, 2008). Plant responses to salinity are complex and a simple and unique ‘solution’ may not exist. Nevertheless, avoiding high Na concentrations in metabolically active compartments of cells is considered essential for tolerance. This is especially important in photosynthetic tissues, and control of Na translocation from root to shoot is crucial. Achieving this goal directly depends on Na transport processes throughout the plant, which include cellular influx/efflux at the root, accumulation in the vacuoles, root to shoot translocation and recirculation via the phloem (Ismail and Horie, 2017).

Although some transporters involved in these processes have been identified, the picture is far from complete (Nieves-Cordones *et al.*, 2016). The transport systems already identified reside in membranes and mediate Na movements across them. However, in some situations, Na could reach the photosynthetic organs through the apoplastic pathway via so-called bypass flow. In rice, a major crop for human nutrition, it has been shown that differences in salinity tolerance can be coupled with differences in the contribution of Na translocation associated with bypass flow (Yeo *et al.*, 1987). Interestingly, the contribution of bypass flow is modulated by silicon (Yeo *et al.*, 1999), an element long neglected by the plant science community (Epstein, 1994) but now shown to be critical in most plant acclimation responses to biotic and abiotic stresses. The precise relationships between the fluxes of Na in the root and bypass flow, and particularly how they are interrelated with Si, remain unclear.

## Cycling of sodium in roots: an emerging hypothesis

According to the prevalent view it is thought that when plants are exposed to high external NaCl concentrations, a large influx of Na occurs towards the root symplast driven by the electrochemical gradient and mediated by low-affinity, non-selective, transport entities (Rains and Epstein, 1965; Davenport and Tester, 2000). As the internal concentration of Na within the root symplast increases, the high Na influx is accompanied by an increased efflux of this cation, which should be against the electrochemical gradient, thus leading to a cycling of Na across the plasma membrane. The work by Flam-Shepherd *et al.* (2018) calls attention to the possible magnitude of the inward and outward fluxes of Na that could take place at the plasma membrane, suggesting that the rapid cycling of Na measured in roots by the commonly used radiometric techniques mainly involves the apoplast.

This possibility had already been advanced by Britto and Kronzucker (2015), who joined sparse pieces of evidence obtained by various authors working with different, and not always comparable, methods. Flam-Shepherd *et al.* (2018) offer a consolidated and unified picture of Na fluxes in the roots of two rice varieties differing in salt tolerance and evaluate the possible incidence of Si on them. Briefly, by performing studies with the ^24^Na radiotracer, the authors report a large influx of Na in both rice varieties, with the efflux of this element unaffected by the presence of a cocktail of respiration inhibitors. They also suggest that, on the basis of electrophysiological recordings, the transmembrane flux of Na may be considerably lower than the influx determined in radiometric studies. Thus, their data suggest that an unknown and probably important fraction of the Na cycled in roots may not involve the plasma membrane.

The authors find further support for their hypothesis in the literature, including the large expenditure of energy necessary to pump out the Na if the efflux of this cation mainly occurs through the plasma membrane; the high Na concentration within the cells that may result from compartmental analysis based on efflux measurements; and the similar half-times for efflux of Na and an apoplastic dye (Britto and Kronzucker, 2009; Kronzucker and Britto, 2011; Coskun *et al.*, 2016). Thus, without diminishing the relevance of Na cycling across the plasma membrane and the transport systems involved, the contribution of apoplastic Na cycling needs to be considered because important consequences may arise:

(i) The large cycling of Na at the apoplast could partially mask the actual cycling of Na at the plasma membrane, thus introducing a significant degree of uncertainty in our current estimations of Na influx and efflux at the plasma membrane, which are mostly based on radiometric studies. This uncertainty could affect a large part of the literature on root Na fluxes.
(ii) The cost of Na pumping out of the cells should be much more reduced than usually thought, which would have major implications for the allocation of plant resources during the acclimation of plants to salinity. A further, related, concept concerns the possibility that a reduced symplastic cycling of Na could operate in organs other than roots, though unfortunately this is probably difficult to establish with commonly used techniques.
(iii) Should the observed differences in Na influx among species and varieties, as determined by radiometric techniques (e.g. comparing values obtained in rice and wheat; see Britto and Kronzucker, 2015 and Santa-María and Epstein, 2001, respectively), be attributed to different amounts of apoplastic Na cycling?
(iv) Could the apoplastic Na cycling act as a signal in responses to salinity?

These are not the only open questions, but they provide an idea of the magnitude of the change in our perception of plant responses to salinity posed by the possibility advanced by the authors (Box 1).

**Box 1. The reduced transmembrane cycling hypothesis and radial transport of sodium in rice**

In this hypothetical scheme, movement of Na from the external medium to the xylem vessels occurs through both symplastic and apoplastic (bypass) radial pathways. The symplastic pathway depends on the net flux of Na at the plasma membrane of outer root cells, which is partially diverted into the vacuoles (Vac) and other root cell compartments. Classically a large influx and efflux of Na has been considered to take place at the plasma membrane, but according to the hypothesis outlined by Flam-Shepherd *et al.* (2018), there must be large Na fluxes in the apoplast, with a reduced contribution via the plasma membrane.

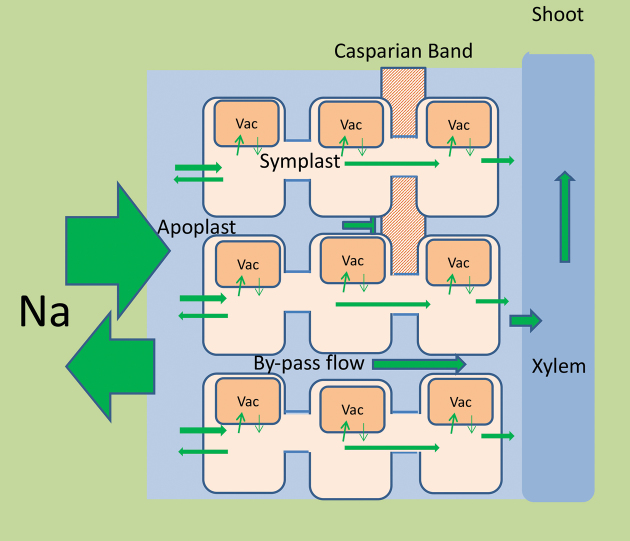

## Alleviation of the effects of salinity by silicon

What about the effect of Si on these fluxes? Rice varieties differ in the bypass flow of Na towards the shoot (Yeo *et al.*, 1987; 1999); for example, in the case considered here, the magnitude of this flow was decreased by Si in the salt-sensitive variety IR29 but not in the salt-tolerant Pokkali. Flam-Shepherd *et al.* (2018) show that this observation was neither accompanied by a differential influence of Si in the cycling of Na in roots nor in its verified transmembrane flux, and so differences in the effect of Si on bypass flow should not be connected with differences in the root Na-cycling process irrespective of whether it takes place in the apoplast or symplast. Thus, effects of Si on plant Na fluxes, at least when the exodermis is not fully developed, are confined to the control of bypass flow. When this significant finding is considered along with previous observations a possible model linking perception of salinity with the protective role of Si seems to emerge (Box 2).

These observations are:

(i) Rice plants exposed to NaCl salinity display enhanced expression of the OsNIP2.1 (Lsi1) transporter, which mediates Si influx to the endodermis and exodermis in the mature root regions; the *lsi1* mutant displays a higher accumulation of Na in the shoot and reduced salt tolerance (Senadheera *et al.*, 2009). This suggests that the action of Si transporters and the deposition of Si in specific tissues (Gong *et al.*, 2006) is required to reduce the delivery of Na to the shoot, which could well be related to the positive effect exerted by Si on the formation of the Casparian Band (Fleck *et al.*, 2011; Fleck *et al.*, 2015).
(ii) In the presence of Si, water loss through transpiration tends to increase (Gong *et al.*, 2006; Flam-Shepherd *et al.*, 2018). This observation could appear contradictory to the role of Si in the formation of the Casparian Band, which should limit the apoplastic flow of water.
(iii) In some plants Si has also been proposed as a main player in determining the activity of aquaporins involved in water transport, particularly under saline conditions and in contributing to osmolyte accumulation, though not by mechanisms which are fully understood (Ríos *et al.*, 2017). Therefore, cycling of Na (or eventually chloride) at the root apoplast and/or the net flux of those ions across the plasma membrane of rice roots could drive a signal leading to the induction of OsNIP2.1, which provides the first step for the cooperative transport of Si (Ma and Yamaji, 2015) and the subsequent chain of events leading to protective effects of Si (Box 2). A possible, and even unexplored, interplay between Si provision and salinization should be considered as the expression of *Lsi1* has been shown to be reduced by Si (Ma and Yamaji, 2015).

**Box 2. The chain of events linking salt fluxes with silicon influx and its beneficial effects**

In this simplified scheme, perception of Na/Cl-cycling in the apoplast and/or net flux of Na/Cl at the plasma membrane lead to enhanced expression of the Si transporter Lsi1, which mediates the influx of Si in the exodermis and endodermis. At least when the exodermis remains undeveloped, Si prevents the accumulation of Na only in the aerial parts of the plant by reducing the contribution of bypass flow at the endodermis, but does not exert any effect on Na cycling in outer root cells. In turn, accumulation of Si positively impacts on water transport through modulation of the activity of aquaporins and accumulation of osmolytes. Broken lines indicate possible regulatory elements but where there is no evidence available.

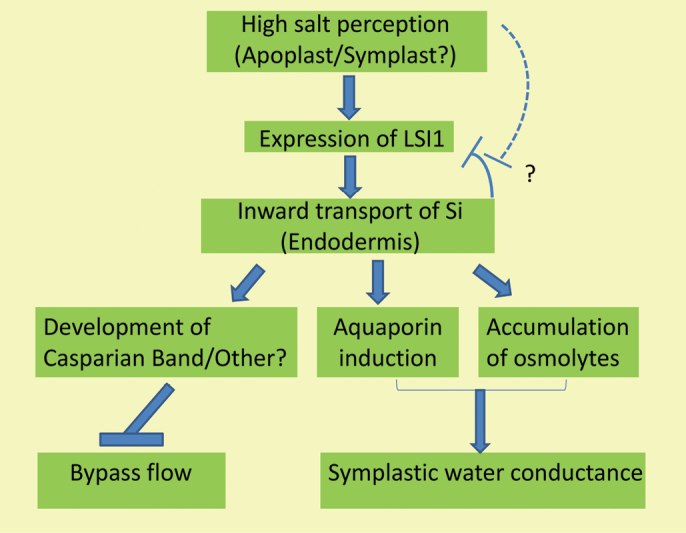

## Conclusions

The work of Flam-Shepherd *et al.* suggests we need to revise our current understanding of the occurrence and magnitude of Na fluxes in the root. It constitutes an excellent starting point for fully exploring the hypothesis that the extent of Na influx and cycling at the plasma membrane is far lower than previously assumed while posing major questions about the relevance of particular unidirectional Na fluxes in the physiological response of plants to salinity, in which Si is increasingly being revealed to be important.
